# Resting‐State Functional Alterations Associated With Endurance Training in Amateur Marathon Runners

**DOI:** 10.1002/brb3.71908

**Published:** 2026-11-10

**Authors:** Fangliang Guo, Houjie Ni, Minghui Zhu, Yushuang Li, Yilin Zhao, Yawen Ao

**Affiliations:** ^1^ Department of Neurology Traditional Chinese and Western Medicine Hospital of Wuhan Tongji Medical College Huazhong University of Science and Technology Wuhan People's Republic of China; ^2^ Department of Radiology Renmin Hospital of Wuhan University Wuhan People's Republic of China

**Keywords:** amateur marathon runners, amplitude of low‐frequency fluctuations, endurance training, functional connectivity, regional homogeneity, resting‐state fMRI

## Abstract

**Background:**

Amateur marathon runners (AMRs) engage in long‐term endurance training in non‐professional contexts, yet the intrinsic brain functional organization associated with amateur marathon training remains unclear. This study examined resting‐state functional alterations in AMRs using regional homogeneity (ReHo), amplitude of low‐frequency fluctuations (ALFF), and seed‐based functional connectivity (FC).

**Methods:**

Sixty‐two AMRs and 42 healthy controls (HCs) underwent resting‐state fMRI. Group differences in mean ReHo (mReHo) and mean ALFF (mALFF) were first examined, followed by seed‐based FC analyses. Within the AMR group, partial correlation and exploratory voxel‐wise correlation analyses were performed to assess associations between imaging measures and behavioral and running‐related variables.

**Results:**

AMRs showed increased mReHo in the left cerebellar lobule VI extending to the lingual gyrus and in the right lingual gyrus, and decreased mReHo in the medial orbitofrontal cortex. They also showed decreased mALFF in the right insula and increased mALFF in the left precuneus extending to the paracentral lobule. Seed‐based FC analyses revealed stronger FC involving medial orbitofrontal‐insula/superior temporal and medial orbitofrontal‐supplementary motor circuits, right lingual‐occipital/cerebellar visual regions, and right insula‐inferior frontal/striatal regions. Within the AMR group, right insula mALFF was positively associated with weekly running volume, while exploratory voxel‐wise analyses additionally identified associations involving the left calcarine cortex, left fusiform gyrus, and left cerebellar Crus I.

**Conclusions:**

AMRs showed distributed resting‐state functional alterations involving visual‐cerebellar, insular, motor‐control, and higher‐order regulatory systems. Given the cross‐sectional design, these findings should be interpreted as associations rather than evidence of training‐induced neural adaptation.

## Graphical abstract

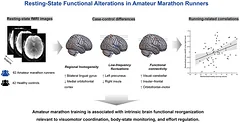

## 1 Introduction

Amateur marathon runners (AMRs) typically train and compete in non‐professional contexts, but they are still exposed to repeated and prolonged physiological demands. Marathon performance relies heavily on aerobic capacity, including maximal oxygen uptake and the ability to sustain a high fraction of this capacity over time (Bassett and Howley 2000; Joyner and Coyle 2008). It is also influenced by running economy, metabolic efficiency, substrate utilization, and thermoregulatory regulation, which together determine how efficiently runners maintain prolonged locomotion (Barnes and Kilding 2015; Foster and Lucia 2007). These physiological demands are closely coupled with brain‐mediated regulatory processes. During long‐distance running, runners must continuously monitor the external environment, maintain posture and spatial orientation, regulate cardiorespiratory and bodily signals, and adjust behavior in response to fatigue, pain, and perceived exertion (Voss et al. 2010). Thus, endurance running depends not only on peripheral physiological capacity, but also on central mechanisms supporting sensory integration, autonomic regulation, cognitive control, and motivation (Hillman et al. 2008). In line with this view, aerobic exercise has been shown to promote brain plasticity through multiple pathways, including neurotrophic signaling, vascular adaptation, synaptic plasticity, and changes in cognitive and large‐scale functional network organization (Cotman and Berchtold 2002; Erickson et al. 2011).

Resting‐state functional magnetic resonance imaging (rs‐fMRI) provides a useful and non‐invasive approach for characterizing intrinsic brain functional organization in AMRs. The blood‐oxygen‐level‐dependent signal measured by fMRI is closely related to underlying neural activity (Logothetis et al. 2001). rs‐fMRI has been widely used to characterize the intrinsic functional architecture of the brain without explicit task demands (Fox and Raichle 2007). Among rs‐fMRI metrics, regional homogeneity (ReHo) measures the local synchronization of spontaneous BOLD signals using Kendall's coefficient of concordance (Zang et al. 2004), whereas amplitude of low‐frequency fluctuations (ALFF) quantifies the intensity of spontaneous low‐frequency neural activity within a given region (Yang et al. 2007). Combining these local measures with seed‐based functional connectivity (FC) may further offer a more comprehensive characterization of both regional and network‐level functional alterations associated with long‐term endurance running. Compared with task‐based fMRI, these approaches are particularly suitable for studying AMRs because they can capture training‐related intrinsic functional characteristics without requiring sport‐specific tasks inside the scanner.

Studies of marathon and ultra‐endurance running have reported that prolonged endurance exercise can induce measurable, and in some cases reversible, brain changes. Ultra‐endurance running has been associated with substantial but reversible reductions in gray matter volume (Freund et al. 2012), while studies of marathon runners have reported acute MRI brain findings and cortical or subcortical volume alterations after marathon or ultramarathon running (Herm et al. 2019; Ozdurak Singin et al. 2021). Marathon running was linked to a reversible reduction in MRI‐derived brain myelin water fraction, suggesting transient changes in white‐matter‐related tissue properties following prolonged endurance exercise (Ramos‐Cabrer et al. 2025). However, these studies mainly focused on structural characteristics or acute post‐race effects. Less is known about the stable intrinsic functional organization associated with long‐term amateur marathon training, particularly in non‐professional runners.

Resting‐state studies have increasingly identified functional differences associated with endurance training. In collegiate distance runners, Raichlen et al. (2016) reported altered connectivity within and between the default mode, frontoparietal, and motor networks, including stronger frontoparietal connectivity with frontal executive regions and greater anticorrelation between the default mode network and paracentral, postcentral, and occipital regions; several of these connectivity measures were also associated with physical activity and estimated aerobic capacity. More recently, S. Yan et al. (2024) found endurance‐training‐related differences in regional activity, FC, and network topology, with ReHo and connectivity alterations involving motor, visual, cerebellar, inferior frontal, and cingulate regions and several measures correlated with training intensity. A recent coordinate‐based meta‐analysis across athletic populations further identified convergent increases in FC in the paracentral lobule, medial frontal gyrus, precuneus, inferior parietal and supramarginal regions, inferior frontal gyrus, Rolandic operculum, and midcingulate cortex, together with increased regional activity in the posterior cingulate, lingual gyrus, and cerebellum (Z. Yan et al. 2025). In addition, de la Cruz et al. (2022) reported stronger resting‐state connectivity within central autonomic and sensorimotor networks in endurance athletes, with connectivity strength inversely associated with heart rate. Collectively, these findings provide prior support for resting‐state differences involving visual‐cerebellar, sensorimotor, and large‐scale control/default‐mode systems in endurance‐trained individuals.

To address this gap, we used rs‐fMRI to investigate spontaneous brain activity and FC in AMRs compared with healthy controls (HCs). Unlike previous studies that primarily examined endurance athletes as a broader group, including competitive or highly trained samples, the present study examined intrinsic resting‐state functional characteristics in non‐professional AMRs using complementary local and connectivity‐based measures. Specifically, we first examined group differences in mReHo and mALFF to characterize local synchronization and regional spontaneous activity intensity, respectively. Brain regions showing significant between‐group differences in mReHo or mALFF were subsequently defined as seed regions for secondary exploratory whole‐brain FC analyses to characterize their broader connectivity patterns. We also explored whether altered neuroimaging measures were associated with behavioral and running‐related variables within the AMR group. Based on prior rs‐fMRI findings in endurance athletes and runners (de la Cruz et al. 2022; Raichlen et al. 2016; S. Yan et al. 2024), we hypothesized that AMRs would show greater local synchronization or spontaneous activity in visual‐cerebellar and sensorimotor regions and altered FC involving visual, motor, and default‐mode systems. Associations with behavioral and running‐related variables were examined exploratorily.

## 2 Methods and Materials

### 2.1 Participants

Participants were recruited through advertisements and local community running clubs from March 2021 to July 2022. All participants were male. All participants provided written informed consent, and the study protocol was approved by the Ethics Committee of Renmin Hospital of Wuhan University (approval no. WDRY2022‐K198). AMR participants were included if they met the following criteria: (1) were not professionally registered athletes, and (2) had engaged in regular endurance training for at least 2 years and completed at least three official half‐marathons. Of the 62 AMRs, 46 had completed at least one full marathon. Detailed information regarding exercise habits, including training duration and the number of completed half‐ and full‐marathons, was collected using self‐report questionnaires. HCs were recruited from the local community and reported no regular engagement in structured exercise or endurance training.

Exclusion criteria for all participants included: (1) a history of head injury with residual effects, (2) neurological or psychiatric disorders, (3) contraindications to MRI, (4) uncontrolled major medical conditions based on self‐report, and (5) a score < 26 on the Beijing version of the Montreal Cognitive Assessment (MoCA‐BJ), using the conventional MoCA screening threshold to exclude participants with possible cognitive impairment (Nasreddine et al. 2005). We recruited a total of 63 AMRs and 43 HCs. One participant in each group was excluded because of incomplete MRI data acquisition. Therefore, a total of 62 AMRs and 42 HCs were included in the final analysis. No prospective sample‐size calculation was performed because the study was based on an existing observational cohort. All participants who met the eligibility criteria and had complete, usable MRI data were included in the final analysis. Detailed demographic and clinical characteristics are summarized in Table 1.

**TABLE 1:** Demographic and characteristics of participants.

Characteristics	AMRs (*n* = 62)	HCs (*n* = 42)	Statistic	Effect size	*p*‐value
Age, years, median (Q1–Q3)	45.5 (39.0–51.0)	41.5 (33.0–52.8)	*U* = 1046	*r*_rb_ = −0.20	0.09
Age range, years	29–59	28–59	—	—	—
Sex, male, *n* (%)	62 (100%)	42 (100%)	—	—	—
Education, years, median (Q1–Q3)	16.0 (15.0–16.8)	16.0 (15.0–17.0)	*U* = 1262	*r*_rb_ = −0.03	0.79
BMI, mean ± SD	23.21 ± 2.09	24.01 ± 2.36	*t* = −1.83	*d* = −0.36	0.07
Mean FD, mm, median (Q1–Q3)	0.0803 (0.0579–0.112)	0.0684 (0.0527–0.0876)	*U* = 1033	*r*_rb_ = −0.21	0.08
Duration of training, years, median (Q1–Q3)	5.0 (3–7)	—	—	—	—
Number of half‐marathons completed, median (Q1–Q3)	10.0 (6.0–29.0)	—	—	—	—
Number of full marathons completed, median (Q1–Q3)	5.5 (0.25–10.8)	—	—	—	—
Weekly running volume, km, median (Q1–Q3)	42.5 (30.0–60.0)	—	—	—	—
PSQI, median (Q1–Q3)	7.0 (5.0–11.0)	9.0 (6.0–13.0)	*U* = 1021	*r*_rb_ = 0.22	0.06
MoCA, median (Q1–Q3)	28.0 (27.0–29.0)	28.0 (27.0–28.8)	*U* = 1280	*r*_rb_ = 0.02	0.88
FSS, mean ± SD	29.69 ± 12.21	33.07 ± 12.89	*t* = −1.35	*d* = −0.27	0.18

*Note*: Data are presented as mean ± SD for normally distributed variables and median (Q1–Q3) for non‐normally distributed variables. Between‐group comparisons were performed using two‐sided independent‐samples *t*‐tests for BMI and FSS and Mann–Whitney *U* tests for age, education, mean FD, PSQI, and MoCA. Effect sizes are reported as Cohen's d for *t*‐tests and rank‐biserial correlation (*r*_rb_) for Mann–Whitney *U* tests.

Abbreviations: AMRs, amateur marathon runners; BMI, body mass index; FD, framewise displacement; FSS, Fatigue Severity Scale; HCs, healthy controls; MoCA, Montreal Cognitive Assessment; PSQI, Pittsburgh Sleep Quality Index.

### 2.2 Behavioral Assessments

For all participants, cognitive performance was assessed using MoCA‐BJ, which has been validated as a screening tool for mild cognitive impairment among Chinese older adults (Yu et al. 2012). The original MoCA was developed as a brief screening tool for detecting mild cognitive impairment (Nasreddine et al. 2005).

Sleep quality was evaluated using the Pittsburgh Sleep Quality Index (PSQI), a validated 19‐item questionnaire that assesses sleep patterns over the previous month (Buysse et al. 1989). The PSQI yields seven component scores, including subjective sleep quality, sleep latency, sleep duration, habitual sleep efficiency, sleep disturbances, use of sleep medication, and daytime dysfunction. A global PSQI score is calculated by summing these components, with higher scores indicating poorer sleep quality.

Fatigue levels were measured using the Fatigue Severity Scale (FSS), a 9‐item self‐report instrument designed for use in the general population. The FSS has demonstrated good internal consistency, high test‐retest reliability, and sensitivity to changes in clinical status (Krupp et al. 1989). Higher FSS scores reflect greater perceived fatigue.

### 2.3 MRI Data Acquisition and Preprocessing

The rs‐fMRI data were acquired at Renmin Hospital of Wuhan University using a 3.0‐T whole‐body MRI scanner with a 16‐channel head coil (Discovery MR750; GE Healthcare, Milwaukee, WI, USA) using a gradient‐echo echo‐planar imaging (GE‐EPI) sequence. The scan parameters were as follows: repetition time (TR) = 2000 ms, echo time (TE) = 30 ms, flip angle = 90°, 38 axial slices, slice thickness = 3.5 mm with no gap, field of view (FOV) = 224 × 224 mm^2^, matrix size = 64 × 64, and isotropic voxel size = 3.5 × 3.5 × 3.5 mm^3^. Each functional session lasted 480 s, resulting in a total of 240 volumes. High‐resolution anatomical images were acquired using a sagittal three‐dimensional T1‐weighted BRAVO sequence with the following parameters: TR/TE = 7.2/2.7 ms, inversion time = 450 ms, flip angle = 12°, 160 slices, slice thickness = 1.0 mm, FOV = 25.6 × 25.6 cm^2^, and matrix = 256 × 256. All scans were visually inspected to exclude gross artifacts. During scanning, participants were instructed to keep their eyes open and fixate, remain still, avoid structured thinking, and not fall asleep. Wakefulness was assessed by self‐report immediately after scanning. All participants were instructed to avoid caffeinated substances and alcoholic drinks for at least 3 days before scanning.

Imaging data were preprocessed using the Data Processing Assistant for Brain Imaging (DPABI, version 5.4; http://rfmri.org/DPARSF) toolbox (C.‐G. Yan et al. 2016) and Statistical Parametric Mapping (SPM12, v7771; http://www.fil.ion.ucl.ac.uk/spm/) implemented in MATLAB R2022b (MathWorks, Natick, MA, USA). Preprocessing procedures included: (1) removal of the first ten time points for signal stabilization, leaving 230 volumes for subsequent analysis; (2) slice‐time correction with the middle slice as the reference frame and realignment to correct for head motion; (3) normalization to MNI space using the EPI template and resampling to 3 × 3 × 3 mm^3^ isotropic voxels; (4) linear detrending and regression of nuisance covariates (including Friston 24 motion parameters and signals of white matter and cerebrospinal fluid). Mean framewise displacement (FD) was calculated according to the Jenkinson definition. Participants were excluded for excessive head motion if maximum translation exceeded 2.5 mm in any direction, maximum rotation exceeded 2.5°, or mean Jenkinson FD exceeded 0.30 mm (Gao et al. 2024). No participants were excluded based on these criteria. Global signal regression and temporal scrubbing were not performed.

### 2.4 ReHo and ALFF Calculation

After preprocessing, ReHo and ALFF were calculated to characterize local synchronization and the intensity of spontaneous neural activity, respectively. For ReHo, the time series were band‐pass filtered at 0.01–0.08 Hz before calculation. Kendall's coefficient of concordance was calculated across each voxel and its 26 neighboring voxels (27‐voxel neighborhood) to quantify local synchronization of spontaneous BOLD activity, generating individual ReHo maps (Zang et al. 2004). For ALFF, the preprocessed time series were transformed into the frequency domain using a fast Fourier transform. The square root of the power spectrum was then calculated, and the mean amplitude within the 0.01–0.08 Hz frequency range was obtained for each voxel as the ALFF value (Yang et al. 2007). The resulting ReHo and ALFF maps were normalized by their respective global mean values to reduce inter‐individual variability, yielding mean ReHo (mReHo) and mean ALFF (mALFF) maps, and were smoothed with a 6‐mm FWHM Gaussian kernel for subsequent group‐level analyses.

### 2.5 Exploratory Seed‐Based FC Analysis

Brain regions showing significant between‐group differences in mReHo or mALFF were defined as regions of interest (ROIs) for secondary exploratory whole‐brain FC analyses. These analyses were intended to characterize broader connectivity patterns associated with regions showing altered local spontaneous activity rather than to provide independent confirmation of the regional findings. For each seed region, the mean time series was extracted from the band‐pass‐filtered and spatially smoothed functional data and correlated with the time series of all other voxels across the brain to generate individual FC maps. The resulting correlation coefficients were transformed to *z*‐scores using Fisher's *r*‐to‐*z* transformation.

### 2.6 Statistical Analysis

Continuous demographic and behavioral variables were assessed for normality using the Shapiro–Wilk test. Normally distributed variables were summarized as mean ± standard deviation (SD) and compared between groups using two‐sided independent‐samples *t*‐tests, whereas variables showing significant deviations from normality were summarized as median (interquartile range, IQR) and compared using Mann–Whitney *U* tests. Specifically, BMI and FSS were analyzed using independent‐samples *t*‐tests, whereas age, education, mean FD, PSQI, and MoCA were analyzed using Mann–Whitney *U* tests. Effect sizes were reported as Cohen's *d* for independent‐samples *t*‐tests and rank‐biserial correlation (*r*_rb_) for Mann–Whitney *U* tests. These analyses were performed using IBM SPSS Statistics, version 27.0 (IBM Corp., Armonk, NY, USA).

Voxel‐wise two‐sample *t*‐tests were conducted to compare whole‐brain mReHo, mALFF, and seed‐based FC maps between the AMRs and HC groups. Age, education, BMI, and mean FD were included as covariates in the between‐group imaging analyses. Sex was not included because all participants were male and therefore there was no between‐subject variation in sex. Statistical analyses were restricted to an AAL‐based brain mask (Tzourio‐Mazoyer et al. 2002). Gaussian random field (GRF) theory was applied to correct for multiple comparisons, with a voxel‐level threshold of *p* < 0.001 and a cluster‐level threshold of *p* 0< 0.05 (two‐tailed).

Partial correlation analyses were conducted within the AMR group to examine associations between mReHo and mALFF extracted from brain regions showing significant between‐group differences and behavioral and running‐related variables, including PSQI, MoCA, FSS scores, duration of training, number of completed half‐ and full‐marathons, and weekly running volume. Age, education, BMI, and FD were included as covariates. Benjamini–Hochberg false discovery rate (FDR) correction was applied separately within each predefined imaging metric. An FDR‐adjusted *p < *0.05 was considered statistically significant.

In addition, exploratory whole‐brain voxel‐wise correlation analyses were also performed by correlating individual mReHo and mALFF maps with behavioral and running‐related variables within AMR group. Age, education, BMI, and FD were included as covariates. Multiple comparisons were corrected using GRF correction, with a voxel‐level threshold of *p < *0.001 and a cluster‐level threshold of *p < *0.05. No additional correction was applied across the 14 whole‐brain maps; therefore, these analyses were considered exploratory and hypothesis‐generating. Anatomical labels were assigned according to the AAL atlas, and for clusters spanning multiple regions, cluster composition was quantified as the number of suprathreshold voxels within each atlas region.

### 2.7 Sensitivity Analyses

Sensitivity analyses included log transformation and extreme‐value exclusion for the skewed half‐marathon count, complementary fractional ALFF (fALFF) analysis, nonparametric permutation testing with threshold‐free cluster enhancement (TFCE) and family‐wise error (FWE) correction of the primary between‐group findings, and additional adjustment for voxel‐wise gray matter volume. Full details are provided in the Supporting Information.

## 3 Results

### 3.1 Demographic and Clinical Characteristics

A total of 62 AMRs and 42 HCs were included in the present study. As shown in Table 1, the AMRs and HC groups did not significantly differ in age, education years, BMI, head motion as indexed by FD, cognitive performance measured by MoCA, PSQI scores, or fatigue level measured by FSS (all *p* > 0.05).

### 3.2 Group Differences in Regional Homogeneity

Compared with HCs, AMRs showed significantly increased mReHo in the left Cerebellum VI extending to lingual gyrus (1863 mm^3^; peak MNI coordinate: *x* = −18, *y* = −75, *z* = −15; peak *t* = 3.775) and in the right lingual gyrus (1485 mm^3^; peak MNI coordinate: *x* = 15, *y* = −60, *z* = −3; peak *t* = 3.436). AMRs showed decreased mReHo in the medial orbitofrontal cortex (1350 mm^3^; peak MNI coordinate: *x* = −3, *y* = 51, *z* = −6; peak *t* = −3.392) (Figure 1A, Table 2).

**FIGURE 1:**
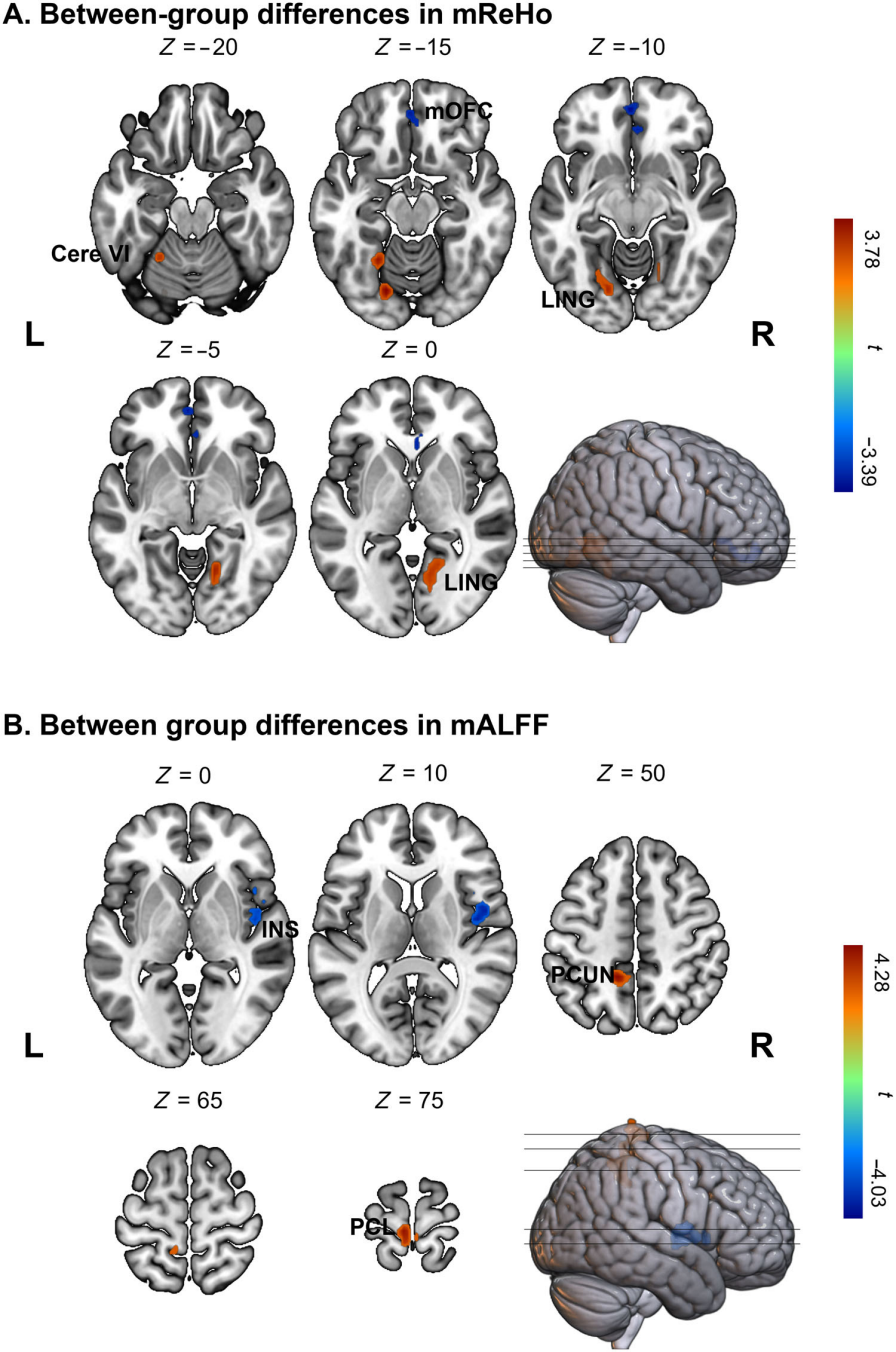
Group differences in mReHo and mALFF between AMRs and HCs. (A) Significant between‐group differences in mReHo. (B) Significant between‐group differences in mALFF. Statistical significance was determined using GRF correction with a voxel‐level threshold of *p* < 0.001 and a cluster‐level threshold of *p *< 0.05 (two‐tailed). The color bar represents *t*‐values, with warm colors indicating higher values in AMRs than HCs. AMRs, amateur marathon runners; Cere VI, Cerebellum VI; HC, healthy controls; INS, insula; LING, lingual gyrus; mALFF, mean amplitude of low‐frequency fluctuations; mOFC, medial orbitofrontal cortex; mReHo, mean regional homogeneity; PCL, paracentral lobule; PCUN, precuneus.

**TABLE 2:** Brain regions showing significant between‐group differences in mReHo and mALFF.

Cluster	Direction	Brain region	Cluster size, mm^3^	Peak MNI coordinate (*x*, *y*, *z*)	Peak *t*‐value	Cluster breakdown (mm^3^)
**mReHo**						
1	AMRs > HCs	Left Cerebellum VI	1863	−18, −75, −15	3.775	Left lingual gyrus (783), left Cerebellum VI (675), left fusiform gyrus (378)
2	AMRs < HCs	Medial orbitofrontal cortex	1350	−3, 51, −6	−3.392	Left medial orbitofrontal cortex (513), right medial orbitofrontal cortex (324), right anterior cingulate cortex (189), left gyrus rectus (135)
3	AMRs > HCs	Right lingual gyrus	1485	15, −60, −3	3.436	Right lingual gyrus (1458)
**mALFF**						
1	AMRs < HCs	Right insula	2484	42, −3, 6	−4.026	Right rolandic operculum (1188), right insula (918), right opercular part of the inferior frontal gyrus (324)
2	AMRs > HCs	Left precuneus	3159	−15, −42, 45	4.227	Left precuneus (1593), left paracentral lobule (837), left midcingulate cortex (189)

*Note*: Statistical significance was determined using GRF correction with a voxel‐level threshold of *p < *0.001 and a cluster‐level threshold of *p <* 0.05. Cluster volumes were calculated based on the normalized voxel size of 3 × 3 × 3 mm^3^ (27 mm^3^ per voxel). Anatomical labeling and cluster composition were based on the AAL atlas. For clarity, the cluster breakdown lists AAL regions containing ≥ 5 suprathreshold voxels (≥ 135 mm^3^); therefore, the listed regional volumes may not sum to the total cluster volume.

Abbreviations: AMRs, amateur marathon runners; GRF, Gaussian random field; HCs, healthy controls; mALFF, mean amplitude of low‐frequency fluctuations; MNI, Montreal Neurological Institute; mReHo, mean regional homogeneity.

### 3.3 Group Differences in mALFF

Compared with HCs, AMRs showed significantly decreased mALFF in the right insula (2484 mm^3^; peak MNI coordinate: *x* = 42, *y* = −3, *z* = 6; peak *t* = −4.026). AMRs showed significantly increased mALFF in the left precuneus extending to the paracentral lobule (3159 mm^3^; peak MNI coordinate: *x* = −15, *y* = −42, *z* = 45; peak *t* = 4.227) (Figure 1B, Table 2).

### 3.4 Seed‐Based Functional Connectivity Differences

Using the medial orbitofrontal cortex cluster showing decreased mReHo as the seed, AMRs showed significantly increased FC with the left insula/superior temporal region (3861 mm^3^; peak MNI coordinate: *x* = −42, *y* = −21, *z* = −9; peak *t* = 4.148) and the supplementary motor area (3753 mm^3^; peak MNI coordinate: *x* = 12, *y* = 0, *z* = 51; peak *t* = 3.533). Using the right lingual gyrus cluster showing increased mReHo as the seed, AMRs showed significantly increased FC with a large bilateral occipital visual cluster involving the lingual gyrus, calcarine cortex, cuneus, and fusiform gyrus, extending to Cerebellum VI (12,339 mm^3^; peak MNI coordinate: *x* = 27, *y* = −60, *z* = −18; peak *t* = 4.237) (Figure 2A, Table 3). Using the right insula cluster showing decreased mALFF as the seed, AMRs showed significantly increased FC with the right triangular part of the inferior frontal gyrus, extending to the right caudate, putamen, and insular regions (3321 mm^3^; peak MNI coordinate: *x* = 45, *y* = 27, *z* = 3; peak *t* = 4.066) (Figure 2B, Table 3). No significant between‐group FC differences were identified for the left lingual/Cerebellum VI or left precuneus/paracentral lobule seeds after GRF correction. In addition, no significant FC decreases in AMRs relative to HCs were observed for any of the five seeds.

**FIGURE 2:**
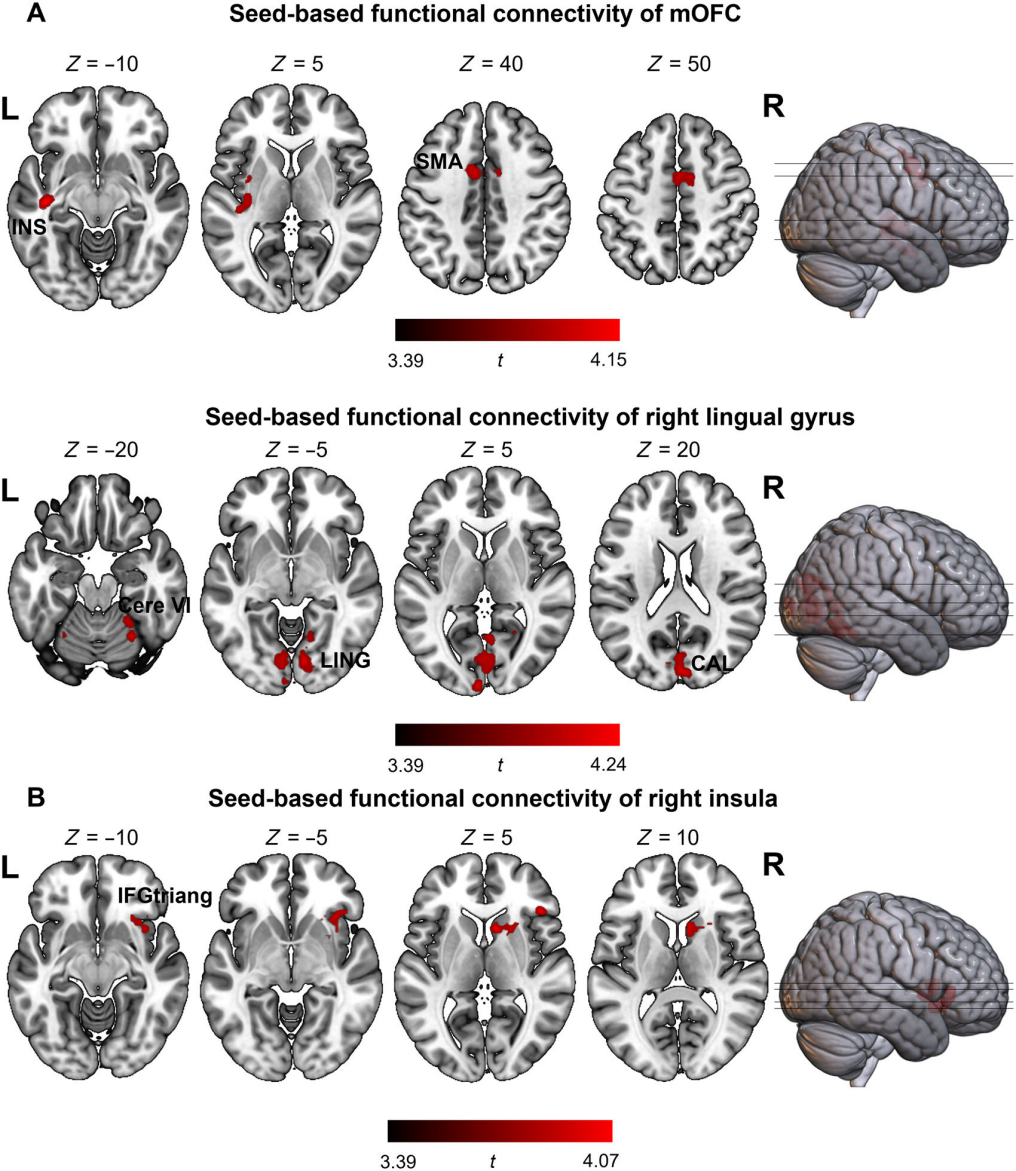
Group differences in seed‐based functional connectivity between AMRs and HCs. (A) Functional connectivity differences using the medial orbitofrontal cortex and right lingual gyrus with significant between‐group differences in mReHo as the seed, respectively. (B) Functional connectivity differences using the right insula showing significant between‐group differences in mALFF as the seed. Statistical significance was determined using GRF correction with a voxel‐level threshold of *p* < 0.001 and a cluster‐level threshold of *p* < 0.05 (two‐tailed). The color bar represents *t*‐values, with warm colors indicating greater functional connectivity in AMRs than HCs. AMRs, amateur marathon runners; CAL, calcarine cortex; Cere VI, Cerebellum VI; FC, functional connectivity; HCs, healthy controls; IFGtriang, triangular part of the inferior frontal gyrus; INS, insula; L, left; LING, lingual gyrus; mALFF, mean amplitude of low‐frequency fluctuations; mReHo, mean regional homogeneity; R, right; SMA, supplementary motor area.

**TABLE 3:** Brain regions showing significant between‐group differences in seed‐based functional connectivity.

Seed‐defining metric	Seed region	Direction	Target region	Cluster size, mm^3^	Peak MNI coordinate (*x*, *y*, *z*)	Peak *t*‐value	Cluster breakdown (mm^3^)
mReHo	Medial orbitofrontal cortex	AMRs > HCs	Left insula	3861	−42, −21, −9	4.148	Left insula (810), left superior temporal gyrus (324), left Heschl's gyrus (297), left middle temporal gyrus (162)
		AMRs > HCs	Bilateral supplementary motor area	3753	12, 0, 51	3.533	Right supplementary motor area (1107), left midcingulate cortex (999), left supplementary motor area (810), right midcingulate cortex (324)
	Right lingual gyrus	AMRs > HCs	Right Cerebellum VI	12,339	27, −60, −18	4.237	Right lingual gyrus (2484), left calcarine cortex (1998), left lingual gyrus (1755), right calcarine cortex (1215), left cuneus (1080), right fusiform gyrus (999), right cuneus (837), left fusiform gyrus (621), right Cerebellum VI (486), left Cerebellum VI (216), left superior occipital gyrus (189), right Cerebellum IV–V (189)
mALFF	Right insula	AMRs > HCs	Right inferior frontal gyrus, triangular part	3321	45, 27, 3	4.066	Right caudate nucleus (972), right insula (783), right putamen (567), right triangular part of the inferior frontal gyrus (405), right orbital part of the inferior frontal gyrus (270), right claustrum (135)

*Note*: Statistical significance was determined using GRF correction with a voxel‐level threshold of *p < *0.001 and a cluster‐level threshold of *p < *0.05. Cluster volumes were calculated based on the normalized voxel size of 3 × 3 × 3 mm^3^ (27 mm^3^ per voxel). Anatomical labeling and cluster composition were based on the AAL atlas. For clarity, the cluster breakdown lists AAL regions containing ≥ 5 suprathreshold voxels (≥ 135 mm^3^); therefore, the listed regional volumes may not sum to the total cluster volume.

Abbreviations: AMRs, amateur marathon runners; FC, functional connectivity; HCs, healthy controls; mALFF, mean amplitude of low‐frequency fluctuations; MNI, Montreal Neurological Institute; mReHo, mean regional homogeneity.

### 3.5 Associations Between Imaging Measures and Running‐Related Variables

Within the AMR group, partial correlation analyses showed that mean mALFF in the right insula was positively associated with weekly running volume (*r* = 0.391, *p = *0.002, FDR‐*p = *0.03; Figure 3A). No other ROI‐level associations survived FDR correction. Fourteen exploratory whole‐brain voxel‐wise analyses were performed (seven behavioral/running‐related variables × two imaging metrics). Positive associations were identified between the number of completed half‐marathons and mALFF in the left calcarine cortex and left fusiform gyrus, and between weekly running volume and mALFF in the left cerebellar Crus I (Table 4, Figure 3B). No clusters showed significant associations between imaging metrics and behavioral/running‐related variables after GRF correction for the remaining 12 maps (Table S2).

**FIGURE 3:**
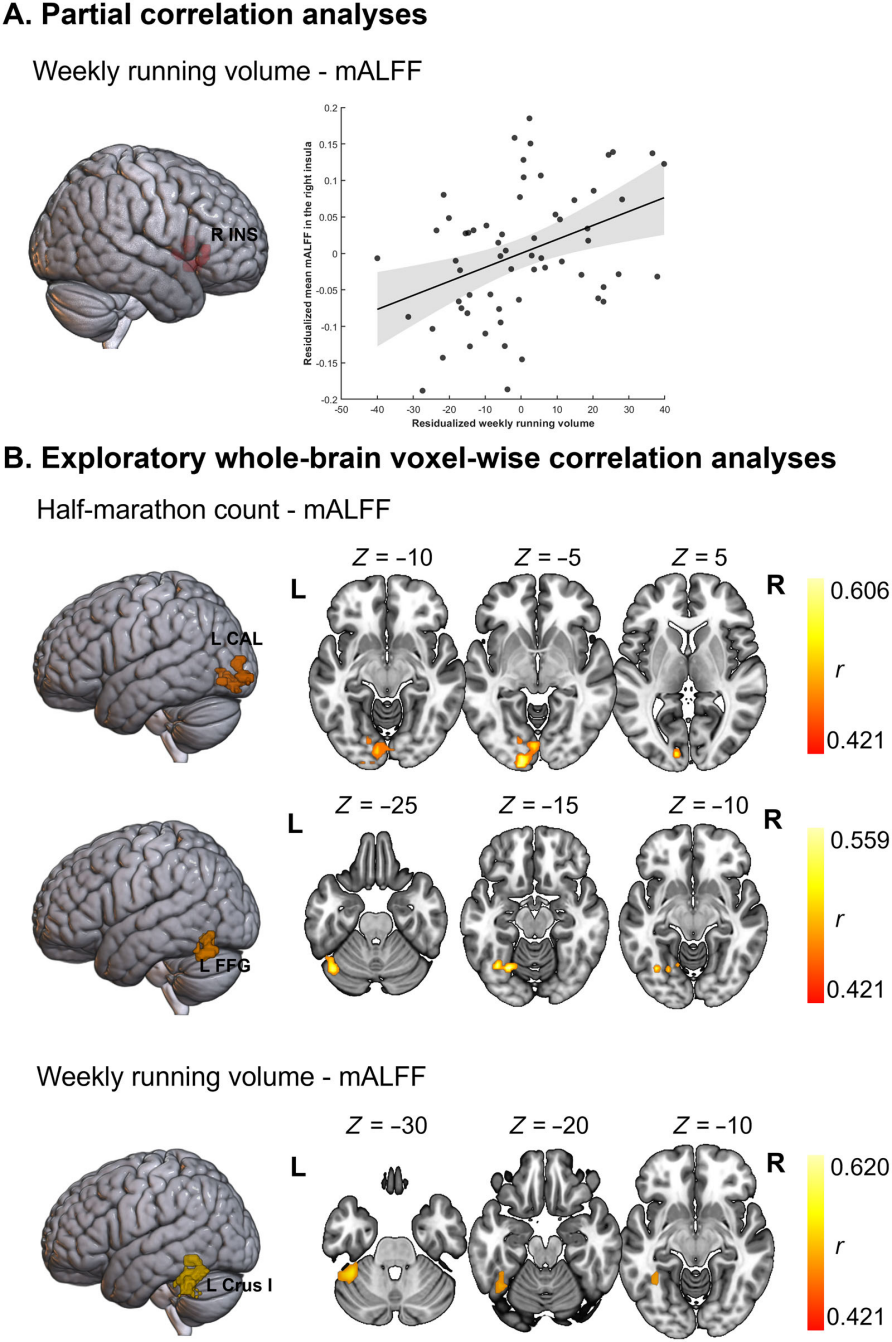
Partial and exploratory correlation analyses within the AMR group. (A) ROI‐level partial correlation analysis showing the association between right insula (R INS) mALFF and weekly running volume. The scatter plot displays residuals and the fitted regression line with the 95% confidence interval. (B) Exploratory whole‐brain voxel‐wise correlation analyses showing positive associations between the number of completed half‐marathons and mALFF in the left calcarine cortex (L CAL) and left fusiform gyrus (L FFG), and between weekly running volume and mALFF in the left cerebellar Crus I (L Crus I). Statistical significance was determined using GRF correction with a voxel‐level threshold of *p* < 0.001 and a cluster‐level threshold of *p* < 0.05 (two‐tailed). Color bars represent voxel‐wise r statistics. AMRs, amateur marathon runners; L, left; mALFF, mean amplitude of low‐frequency fluctuations; R, right; ROI, region of interest.

**TABLE 4:** Brain regions showing significant voxel‐wise correlations between mALFF and running‐related variables in amateur marathon runners.

Variable	Peak brain region	Cluster size, mm^3^	Peak MNI coordinate (*x*, *y*, *z*)	Peak *r* statistic	Cluster breakdown (mm^3^)
Half‐marathon count	Left calcarine cortex	3942	−15, −96, −3	0.607	Left calcarine cortex (1701), left lingual gyrus (1026), left superior occipital gyrus (432), left middle occipital gyrus (243), right lingual gyrus (216), left inferior occipital gyrus (162), right calcarine cortex (162)
	Left fusiform gyrus	2916	−36, −66, −12	0.559	Left cerebellar Crus I (1080), left fusiform gyrus (972), left Cerebellum VI (459), left lingual gyrus (297)
Weekly running volume	Left cerebellar Crus I	4320	−39, −45, −32	0.62	Left cerebellar Crus I (621), left Cerebellum VI (378), left fusiform gyrus (297)

*Note*: Only significant voxel‐wise associations surviving GRF correction are shown. Peak *r* statistics represent voxel‐wise correlation statistics from selected suprathreshold clusters and should not be interpreted as unbiased effect‐size estimates. Cluster volumes were calculated based on the normalized voxel size of 3 × 3 × 3 mm^3^ (27 mm^3^ per voxel). Anatomical labeling and cluster composition were based on the AAL atlas.

Abbreviations: AMR, amateur marathon runner; mALFF, mean amplitude of low‐frequency fluctuations; MNI, Montreal Neurological Institute.

### 3.6 Results of Sensitivity Analyses

Sensitivity analyses showed mixed robustness of the primary findings. The original left calcarine and fusiform associations with half‐marathon count were not reproduced at the prespecified corrected threshold after either log transformation or exclusion of extreme observations; only partly overlapping posterior cerebellar/fusiform effects were observed descriptively at an uncorrected threshold and therefore should be interpreted with caution (Figure S1; Table S1). In the complementary fALFF analysis, AMRs showed significantly higher mean fALFF in a medial occipital cluster centered on the left calcarine cortex (3105 mm^3^; peak MNI coordinate: 0, −84, 12; peak *t = *3.461; Figure S2). None of the primary GRF‐significant between‐group findings survived permutation‐based TFCE‐FWE correction at *p *< 0.05. Likewise, after additional adjustment for voxel‐wise gray matter volume, no mReHo or mALFF group differences survived the prespecified multiple‐comparison correction, although some spatially overlapping effects were observed descriptively at an uncorrected threshold (Figure S3).

## 4 Discussion

In the present study, we investigated resting‐state functional alterations in AMRs using complementary measures of local spontaneous activity and seed‐based FC. Compared with HCs, AMRs showed altered local spontaneous activity in visual‐cerebellar and higher‐order regulatory regions, including increased mReHo in the left cerebellar lobule VI extending to the lingual gyrus and in the right lingual gyrus, decreased mReHo in the medial orbitofrontal cortex, decreased mALFF in the right insula, and increased mALFF in the left precuneus extending to the paracentral lobule. Exploratory seed‐based analyses further revealed stronger FC in AMRs, including mOFC connectivity with the left insula/superior temporal region and supplementary motor area, connectivity of right lingual gyrus with a large bilateral occipital‐cerebellar visual cluster, and right insula connectivity with the right inferior frontal gyrus triangular part extending to striatal and insular regions. In addition, right insula mALFF was positively associated with weekly running volume, while exploratory voxel‐wise analyses additionally identified associations involving the left calcarine cortex, left fusiform gyrus, and left cerebellar Crus I. Together, these findings indicate distributed resting‐state functional differences across visual‐cerebellar, insular, motor‐control, and higher‐order regulatory systems in AMRs.

The first key finding was the involvement of visual‐cerebellar systems in AMRs. Long‐distance running requires continuous monitoring of the external environment, adjustment to terrain, maintenance of spatial orientation, and online visuomotor regulation of gait and posture. The involvement of the calcarine cortex, lingual gyrus, and fusiform gyrus is therefore biologically plausible, as these regions support early visual processing, higher‐order visual analysis, and visuospatial integration (Palejwala et al. 2020). Beyond its classical role in motor coordination, the cerebellum contributes to predictive control, timing, sensorimotor integration, and cognitive regulation through its interactions with cerebral association networks (Buckner 2013; Buckner et al. 2011). Although Cerebellum VI is often linked to motor coordination and locomotor control, cerebellar Crus I is more strongly connected with association networks and has been implicated in cognitive, affective, and predictive processes (Buckner et al. 2011; Schmahmann and Sherman 1998; S. Yan et al. 2024). Thus, the involvement of occipital visual cortices and cerebellar regions is broadly consistent with their established roles in visual processing, sensorimotor integration, timing, and predictive control. However, the present resting‐state data do not directly establish their involvement in specific endurance‐related processes such as pacing or effort regulation. This interpretation is consistent with prior studies of endurance athletes reporting altered regional activity and FC in motor, visual, and cerebellar regions, with some measures associated with training intensity (S. Yan et al. 2024), and with a recent athlete rs‐fMRI meta‐analysis identifying convergent alterations in the lingual gyrus and cerebellum (Z. Yan et al. 2025). The exploratory associations between running exposure and mALFF in visual and cerebellar regions provide additional evidence that these functional characteristics may vary with running exposure, although these associations should be interpreted cautiously given the cross‐sectional design and the limited robustness of the half‐marathon findings.

Another key finding is the involvement of the right insula and its connectivity with the inferior frontal and striatal regions. Although AMRs showed lower mALFF in the right insula than HCs, higher right insula mALFF was associated with greater weekly running volume within the AMR group. These findings should not be regarded as convergent evidence, because between‐group differences and within‐group exposure‐related associations reflect distinct statistical contrasts. The opposite directions may reflect heterogeneity in training exposure, possible non‐linear relationships, or residual confounding, and cannot be resolved within the present cross‐sectional design. In parallel, AMRs showed stronger right insula‐based FC with the right triangular part of the inferior frontal gyrus, extending to striatal and insular regions. This pattern suggests that local spontaneous activity and long‐range network coupling may capture different aspects of insular functional organization. The insula is a central hub for interoception, autonomic regulation, salience detection, and switching between large‐scale brain networks (Menon and Uddin 2010), all of which are relevant to endurance exercise. The inferior frontal gyrus and insula are also involved in attentional and cognitive‐control processes (Tops and Boksem 2011). Greater insula–IFG connectivity in AMRs therefore indicates altered functional coupling between these regions, although its specific behavioral significance for effort evaluation, discomfort regulation, or pacing cannot be established from the present resting‐state data. This interpretation is broadly consistent with endurance‐performance models emphasizing the contribution of perceived effort and central processes to endurance performance (Marcora et al. 2009; Pageaux 2014; St Clair Gibson et al. 2003). Moreover, de la Cruz et al. (2022) reported central autonomic network alterations in male endurance athletes, including insula‐related autonomic and sensorimotor circuits. Overall, the present findings suggest involvement of insular and frontostriatal functional systems in AMRs, but their specific functional and training‐related significance requires further investigation.

AMRs also showed alterations in higher‐order regulatory and integrative systems, involving the mOFC, precuneus, paracentral lobule, and SMA. Although AMRs showed decreased mReHo in the mOFC, mOFC‐seeded FC was stronger with the left insula/superior temporal region and the SMA. The orbitofrontal cortex has established roles in reward valuation, affective evaluation, outcome monitoring, and decision‐making (Rolls 2023; Rolls and Grabenhorst 2008). Although these functions are relevant to motivated behavior, the present resting‐state findings do not directly demonstrate involvement of the mOFC in effort valuation, motivation, or training adherence. Greater mOFC connectivity with the insula/superior temporal region and SMA indicates differences in functional coupling among regions involved in sensory/interoceptive processing and motor‐related functions, although the behavioral significance of these differences remains uncertain. The SMA is involved in motor planning and internally generated movement (Nachev et al. 2008; Shackman et al. 2011). In parallel, increased mALFF in the left precuneus extending to the paracentral lobule suggests altered spontaneous activity in regions supporting default‐mode, visuospatial, and body‐related integration. The precuneus is a central hub of the default mode network and is linked to self‐referential processing, visuospatial imagery, episodic memory retrieval, and integration of internal and external information (Cavanna and Trimble 2006; Utevsky et al. 2014), while the adjacent paracentral lobule contains medial sensorimotor representations relevant to lower‐limb movement and body‐state processing. Together, these findings indicate resting‐state functional differences involving higher‐order and sensorimotor‐related regions, but their specific behavioral significance requires direct testing.

Several limitations should be acknowledged. First, the cross‐sectional design precludes causal inference, and the observed group differences may partly reflect pre‐existing characteristics rather than effects of endurance training. Second, all participants were male, limiting generalizability to female runners, and handedness, smoking status, habitual alcohol and caffeine consumption, and time since the last training session were not systematically available. Third, running‐related variables were based on self‐report, and objective measures of cardiorespiratory fitness and performance (e.g., VO_2_max, lactate threshold, resting heart rate, and verified race times) were not available. Physical activity outside formal endurance training was also not quantified using a validated instrument, limiting characterization of habitual activity, particularly in HCs. Fourth, physiological and vascular factors that may influence BOLD signals, including heart‐rate variability, respiration, cerebral blood flow, and cerebrovascular reactivity, were not measured. Other lifestyle factors may also contribute to residual variability; notably, PSQI scores were relatively elevated in both groups despite no significant between‐group difference after reanalysis. Fifth, because FC seeds were defined from regions showing group differences in the same sample, the seed‐based analyses were not statistically independent and should be considered secondary and hypothesis‐generating. Finally, several significant clusters were relatively small, and the exploratory voxel‐wise associations and primary GRF‐significant findings showed limited robustness in sensitivity analyses, including permutation‐based TFCE‐FWE correction. The absence of an independent replication sample further limits confidence in the reproducibility of these findings. Replication in larger, independent, and longitudinal cohorts is therefore needed.

In conclusion, AMRs showed distributed resting‐state functional differences involving visual‐cerebellar, insular, motor‐control, and higher‐order regulatory systems. Associations with running‐related variables provide preliminary evidence that some of these functional characteristics may vary with running exposure, although the exploratory nature and limited robustness of several findings warrant caution. Given the cross‐sectional design, these results should not be interpreted as evidence of training‐induced neural adaptation or specific behavioral mechanisms. Future longitudinal studies incorporating objective measures of cardiorespiratory fitness, physiological monitoring, and independent replication are needed to clarify their functional and training‐related significance.

## Author Contributions

**Fangliang Guo**: conceptualization, investigation, data curation, formal analysis, visualization, writing – original draft, funding acquisition. **Houjie Ni**: conceptualization, investigation, data curation, formal analysis, visualization, writing – original draft. **Minghui Zhu**: conceptualization, investigation, data curation, formal analysis, visualization, writing – original draft. **Yushuang Li**: investigation, data curation, MRI data acquisition, quality control. **Yilin Zhao**: investigation, data curation, MRI data acquisition, quality control. **Yawen Ao**: conceptualization, methodology, supervision, project administration, funding acquisition, writing – review and editing. All authors have read and approved the final version of the manuscript.

## Funding

This work was supported by the National Natural Science Foundation of China (Y.W.A., Grant No. 82302868), the Natural Science Foundation of Wuhan (F.L.G., Grant No. 2025020701020232) and the Hubei Provincial Natural Science Foundation of China (F.L.G., Grant No. 2026AFB451). The funders had no role in the study design, data collection, data analysis, interpretation of data, decision to publish, or preparation of the manuscript.

## Disclosure

Yawen Ao, as the manuscript guarantor, had full access to all of the data in this study and takes complete responsibility for the integrity of the data and the accuracy of the data analysis.

## Ethics Statement

All participants provided written informed consent, and the study protocol was approved by the Ethics Committee of Renmin Hospital of Wuhan University (approval no. WDRY2022‐K198).

## Conflicts of Interest

The authors declare no conflicts of interest.

## Supporting information




**Supplementary Material**: brb371908‐sup‐0001‐SuppMat.docx

## Data Availability

The datasets analyzed during the current study are not publicly available because of participant privacy and ethical restrictions but are available from the corresponding author on reasonable request.
