# Supplementary material for: Resting‐State Functional Alterations Associated With Endurance Training in Amateur Marathon Runners
Source: Brain Behav. 2026 Nov 10;16(11):e71908. doi: 10.1002/brb3.71908 (PMC13750108; doi:10.1002/brb3.71908)
Supplement: Supplementary file 1 [file BRB3-16-e71908-s001.docx]

**Supplementary Methods**

**Sensitivity analyses**

Several complementary sensitivity analyses were conducted to assess the robustness of the primary findings. First, because the number of completed half-marathons was markedly right-skewed, the exploratory voxel-wise analysis was repeated using ln(1 + Half-marathon count) in the full AMR sample (n = 62) and after excluding participants with values above the upper Tukey fence (Q3 + 1.5 × IQR; >63.5 completed half-marathons; n = 8). Second, fractional ALFF (fALFF) was examined as a complementary measure of low-frequency BOLD fluctuations, and between-group mean fALFF differences were assessed using the same covariates and multiple-comparison correction as in the primary mALFF analysis. Third, the primary between-group imaging analyses were repeated using nonparametric permutation testing with threshold-free cluster enhancement (TFCE) and family-wise error (FWE) correction at *P < .*05 to evaluate the robustness of the GRF-based findings to an alternative multiple-comparison procedure. Fourth, to assess whether regional structural variation could contribute to the observed functional differences, the between-group mReHo and mALFF analyses were repeated with voxel-wise grey matter volume included as an additional covariate, together with age, education, BMI, and mean FD. The same statistical framework as in the primary analyses was applied. Findings not surviving the prespecified multiple-comparison correction were examined descriptively at an uncorrected voxel-level threshold of *P < .*05.

**Supplementary Results**

**Results of the sensitivity analyses**

The sensitivity analyses yielded mixed evidence regarding the robustness of the primary findings. For the half-marathon count analysis, neither the log-transformed analysis nor the analysis excluding eight participants with extreme values reproduced the original left calcarine and left fusiform associations at the prespecified multiple-comparison-corrected threshold. At an uncorrected voxel-level threshold of *P < .*05, the log-transformed analysis identified a cluster peaking in the left cerebellar Crus I (3996 mm³; MNI: −42, −57, −30; peak *r* = 0.459), extending mainly into the left cerebellar lobule VI and left fusiform gyrus. After exclusion of the eight extreme observations, a partly overlapping cluster peaking in the left cerebellar lobule VI was observed (4158 mm³; MNI: −30, −60, −24; peak *r* = 0.511), with extension into the left cerebellar Crus I and fusiform gyrus. These uncorrected findings are presented descriptively in Figure S1 and Table S1 and indicate that the original voxel-wise associations with half-marathon count were sensitive to the skewed exposure distribution and extreme observations.

In the complementary fALFF analysis, AMRs showed significantly higher mean fALFF in a medial occipital cluster centered on the left calcarine cortex (3105 mm³; peak MNI coordinate: 0, −84, 12; peak *t* = 3.461), with predominant involvement of the left calcarine cortex and left cuneus and smaller extensions into the right calcarine cortex and right cuneus (Figure S2).

The primary GRF-significant between-group findings did not survive permutation-based TFCE-FWE correction at *P < .*05, indicating that these findings were sensitive to the choice of multiple-comparison procedure and should therefore be interpreted cautiously.

At an uncorrected voxel-level threshold of *P < .*05, the mReHo analysis showed clusters involving the left cerebellum/lingual region, medial orbitofrontal/anterior cingulate region, medial superior frontal gyrus, right superior temporal pole, and posterior cerebellum. The mALFF analysis showed clusters involving the left precuneus/paracentral region and the right postcentral–precentral region. Some of these effects spatially overlapped with the primary findings, particularly the left cerebellar–lingual and left precuneus/paracentral clusters (Figure S3); however, none remained significant after correction. These findings therefore indicate limited robustness of the functional differences to adjustment for regional grey matter volume.

**Figure S1. Sensitivity analyses of voxel-wise associations with half-marathon count.**

**Figure S1. Sensitivity analyses of exploratory voxel-wise associations with half-marathon count.** (A) Whole-brain voxel-wise analysis using ln(1 + half-marathon count) in the full AMR sample. (B) Whole-brain voxel-wise analysis using the original half-marathon count after excluding eight participants with values above the upper Tukey fence. Color bars represent voxel-wise correlation statistics. Abbreviations: AMR, amateur marathon runner; GRF, Gaussian random field.

**Figure S2. Sensitivity analysis of between-group differences in fALFF between amateur marathon runners and healthy controls.** AMRs showed significantly higher fALFF in a medial occipital cluster centered on the left calcarine cortex (CAL), with extension into the bilateral calcarine cortex and cuneus (CUN). Statistical maps were thresholded using the same multiple-comparison correction applied in the primary mALFF analysis. Color bars represent *t* statistics. Abbreviations: AMRs, amateur marathon runners; fALFF, fractional amplitude of low-frequency fluctuations; HCs, healthy controls; MNI, Montreal Neurological Institute.

**Figure S3. Sensitivity analysis adjusting for voxel-wise grey matter volume.** Between-group differences in (A) mReHo and (B) mALFF after additionally including voxel-wise grey matter volume as a covariate, together with age, education, BMI, and mean FD. For descriptive purposes, maps are displayed at an uncorrected voxel-level threshold of *P < .*05. In panel A, warm colors indicate higher and cool colors indicate lower mReHo values in AMRs relative to HCs. In panel B, warm colors indicate higher mALFF values in AMRs relative to HCs. Color bars represent voxel-wise *t* statistics.

**Table S1. Sensitivity analyses of exploratory voxel-wise associations between mALFF and half-marathon count in amateur marathon runners.**

| **Variable** | **AMR sample size, n** | **Peak brain region** | **Cluster size, mm³** | **Peak MNI coordinate (*x*, *y*, *z*)** | **Peak *r* statistic** | **Cluster breakdown (mm³)** |
| --- | --- | --- | --- | --- | --- | --- |
| ln(1 + Half-marathon count) | 62 | Left cerebellar Crus I | 3996 | -42, -57, -30 | 0.459 | Left cerebellar Crus I (2322), left cerebellar lobule VI (837), left fusiform gyrus (702), left inferior temporal gyrus (135) |
| Original half-marathon count with extreme-value exclusion | 54 | Left cerebellar lobule VI | 4158 | -30, -60, -24 | 0.511 | Left cerebellar Crus I (2376), left cerebellar lobule VI (1215), left fusiform gyrus (432), left inferior temporal gyrus (135) |

**Note:** The clusters shown here were observed at an uncorrected voxel-level threshold of *P* < .05 and did not survive the prespecified multiple-comparison correction. Peak *r* statistics represent voxel-wise correlation statistics and should not be interpreted as unbiased effect-size estimates. Cluster volumes were calculated based on the normalized voxel size of 3 × 3 × 3 mm³ (27 mm³ per voxel). Anatomical labeling and cluster composition were based on the AAL atlas.

**Abbreviations:** AAL, Automated Anatomical Labeling; AMR, amateur marathon runner; BMI, body mass index; FD, framewise displacement; mALFF, mean amplitude of low-frequency fluctuations; MNI, Montreal Neurological Institute.

**Table S2. Summary of exploratory whole-brain voxel-wise associations between resting-state measures and behavioral and running-related variables in amateur marathon runners.**

| **Imaging metric** | **Variable** | **Whole-brain result after GRF correction** |
| --- | --- | --- |
| mReHo | PSQI | No significant clusters |
|  | MoCA | No significant clusters |
|  | FSS | No significant clusters |
|  | Training duration | No significant clusters |
|  | Half-marathon count | No significant clusters |
|  | Full-marathon count | No significant clusters |
|  | Weekly running volume | No significant clusters |
| mALFF | PSQI | No significant clusters |
|  | MoCA | No significant clusters |
|  | FSS | No significant clusters |
|  | Training duration | No significant clusters |
|  | Half-marathon count | Significant: left calcarine cortex and left fusiform gyrus |
|  | Full-marathon count | No significant clusters |
|  | Weekly running volume | Significant: left cerebellar Crus I |

**Abbreviations:** FSS, Fatigue Severity Scale; GRF, Gaussian random field; mALFF, mean amplitude of low-frequency fluctuations; MoCA, Montreal Cognitive Assessment; mReHo, mean regional homogeneity; PSQI, Pittsburgh Sleep Quality Index.

**Table S3. Partial correlations between resting-state imaging measures and behavioral and running-related variables in amateur marathon runners.**

| **Brain regions** | **Variable** | **Partial *r* value** | **Raw *P* value** | **FDR-*P* value** |
| --- | --- | --- | --- | --- |
| **mReHo** | | | | |
| Left Cerebellum VI | Duration of training | -0.023 | 0.87 | 0.97 |
|  | Number of half-marathons completed | 0.275 | 0.04 | 0.64 |
|  | Number of full marathons completed | -0.087 | 0.52 | 0.95 |
|  | Weekly running volume | 0.184 | 0.17 | 0.64 |
|  | PSQI | -0.213 | 0.11 | 0.64 |
|  | FSS | -0.101 | 0.45 | 0.95 |
|  | MoCA | -0.029 | 0.83 | 0.97 |
| Medial orbitofrontal cortex | Duration of training | -0.222 | 0.10 | 0.64 |
|  | Number of half-marathons completed | -0.160 | 0.23 | 0.69 |
|  | Number of full marathons completed | 0.040 | 0.77 | 0.97 |
|  | Weekly running volume | 0.060 | 0.65 | 0.97 |
|  | PSQI | 0.130 | 0.33 | 0.87 |
|  | FSS | -0.006 | 0.96 | 0.98 |
|  | MoCA | -0.005 | 0.97 | 0.98 |
| Right lingual gyrus | Duration of training | 0.177 | 0.18 | 0.64 |
|  | Number of half-marathons completed | 0.198 | 0.14 | 0.64 |
|  | Number of full marathons completed | 0.056 | 0.68 | 0.97 |
|  | Weekly running volume | 0.092 | 0.49 | 0.95 |
|  | PSQI | 0.082 | 0.54 | 0.95 |
|  | FSS | 0.049 | 0.71 | 0.97 |
|  | MoCA | 0.016 | 0.90 | 0.97 |
| **mALFF** | | | | |
| Right insula | Duration of training | 0.014 | 0.92 | 0.96 |
|  | Number of half-marathons completed | 0.079 | 0.56 | 0.96 |
|  | Number of full marathons completed | -0.018 | 0.89 | 0.96 |
|  | Weekly running volume | 0.391 | 0.00 | 0.03 |
|  | PSQI | -0.077 | 0.56 | 0.96 |
|  | FSS | -0.029 | 0.83 | 0.96 |
|  | MoCA | 0.077 | 0.56 | 0.96 |
| Left precuneus | Duration of training | -0.136 | 0.31 | 0.96 |
|  | Number of half-marathons completed | 0.059 | 0.66 | 0.96 |
|  | Number of full marathons completed | -0.151 | 0.26 | 0.96 |
|  | Weekly running volume | 0.041 | 0.76 | 0.96 |
|  | PSQI | 0.006 | 0.96 | 0.96 |
|  | FSS | 0.019 | 0.89 | 0.96 |
|  | MoCA | -0.112 | 0.40 | 0.96 |

**Note:** Benjamini–Hochberg false discovery rate (FDR) correction was applied separately within each predefined imaging metric: 21 tests for mReHo (3 ROIs × 7 variables) and 14 tests for mALFF (2 ROIs × 7 variables). FDR-adjusted *P* < .05 was considered statistically significant.
**Abbreviations:** BMI, body mass index; FD, framewise displacement; FDR, false discovery rate; FSS, Fatigue Severity Scale; mALFF, mean amplitude of low-frequency fluctuations; MoCA, Montreal Cognitive Assessment; mReHo, mean regional homogeneity; PSQI, Pittsburgh Sleep Quality Index.
